# CardioNet: Automatic Semantic Segmentation to Calculate the Cardiothoracic Ratio for Cardiomegaly and Other Chest Diseases

**DOI:** 10.3390/jpm12060988

**Published:** 2022-06-17

**Authors:** Abbas Jafar, Muhammad Talha Hameed, Nadeem Akram, Umer Waqas, Hyung Seok Kim, Rizwan Ali Naqvi

**Affiliations:** 1 Department of Computer Engineering, Myongji University, Yongin 03674, Korea; jafarabbas1272@gmail.com; 2 Department of Primary and Secondary Healthcare, Lahore 54000, Pakistan; talha.ghauri786@gmail.com (M.T.H.); shona4488059@gmail.com (N.A.); 3 Research and Development, AItheNutrigene, Seoul 06132, Korea; umer@aithenutrigene.com; 4 School of Intelligent Mechatronics Engineering, Sejong University, Seoul 05006, Korea; 5 Department of Unmanned Vehicle Engineering, Sejong University, Seoul 05006, Korea

**Keywords:** cardiothoracic ratio, transverse cardiac diameter, semantic segmentation, CardioNet, chest anatomy

## Abstract

Semantic segmentation for diagnosing chest-related diseases like cardiomegaly, emphysema, pleural effusions, and pneumothorax is a critical yet understudied tool for identifying the chest anatomy. A dangerous disease among these is cardiomegaly, in which sudden death is a high risk. An expert medical practitioner can diagnose cardiomegaly early using a chest radiograph (CXR). Cardiomegaly is a heart enlargement disease that can be analyzed by calculating the transverse cardiac diameter (TCD) and the cardiothoracic ratio (CTR). However, the manual estimation of CTR and other chest-related diseases requires much time from medical experts. Based on their anatomical semantics, artificial intelligence estimates cardiomegaly and related diseases by segmenting CXRs. Unfortunately, due to poor-quality images and variations in intensity, the automatic segmentation of the lungs and heart with CXRs is challenging. Deep learning-based methods are being used to identify the chest anatomy segmentation, but most of them only consider the lung segmentation, requiring a great deal of training. This work is based on a multiclass concatenation-based automatic semantic segmentation network, CardioNet, that was explicitly designed to perform fine segmentation using fewer parameters than a conventional deep learning scheme. Furthermore, the semantic segmentation of other chest-related diseases is diagnosed using CardioNet. CardioNet is evaluated using the JSRT dataset (Japanese Society of Radiological Technology). The JSRT dataset is publicly available and contains multiclass segmentation of the heart, lungs, and clavicle bones. In addition, our study examined lung segmentation using another publicly available dataset, Montgomery County (MC). The experimental results of the proposed CardioNet model achieved acceptable accuracy and competitive results across all datasets.

## 1. Introduction

The most used and evaluated method of diagnosing chest-related pathologies such as pneumothorax, pulmonary cancer, congestive heart failure, lung nodule, and heart enlargement is the chest X-ray (CXRs) [1]. Heart enlargement is classified as cardiomegaly, one of the serious cardiovascular diseases among the general public [2]. Cardiomegaly can happen from different conditions such as cardiac insufficiency, blood pressure, hypertension, and coronary artery disease. These cardiac concerns affect patients’ health ranging from a high risk of heart failure to immediate death [3]. Therefore, the early diagnosis of cardiomegaly is critical; the disease can be diagnosed with edge detection of the size and shape of the chest and heart from posterior–anterior (PA) CXR. The cardiothoracic ratio (CTR) is a quantitative measure of heart enlargements in CXRs to detect cardiomegaly and the boundaries of other chest organs [4,5].

**Figure 1:**
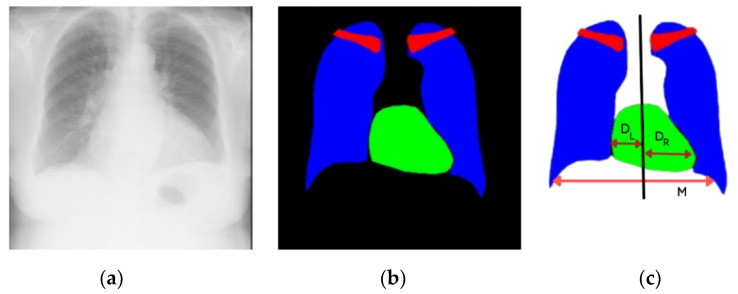
Chest anatomy segmentation to calculate cardiomegaly: (**a**) original CXR PA image, (**b**) segmented image by CardioNet, (**c**) maximum width of heart and thorax to calculate the CTR.

The CTR is the ratio between the maximum horizontal cardiac diameter and the maximum horizontal thoracic diameter, and the normal range is between 0.42 and 0.50. A value higher than normal (>0.50) is considered cardiomegaly [5]. The manual measurement of the CTR from CXR performed by medical experts requires the domain knowledge of chest physiologies. Cardiologists detect cardiomegaly by measuring the heart’s left distance, $D_{L}$, and right distance, $D_{R}$, boundaries from the central vertical line of the chest. M is the maximum horizontal distance between the left- and right-side boundaries of the respective lungs, as shown in Figure 1. The method of calculating the CTR for cardiomegaly is expressed in Equation (1). There is the possibility of observational error, and the process is time-consuming. This problem has motivated researchers to develop a computer-aided diagnosis- (CAD) based CTR measurement to diagnose cardiomegaly. Several researchers have automatically measured the CTR and other heart diseases [6,7]. Most of the techniques used to detect the boundaries and size of lungs and heart require the accurate segmentation of anatomical organs.

(1) $$\mathrm{CTR}=\left(D_{L}+D_{R}\right)/M$$

In medical imaging, segmentation is extracted with similar properties from the images. The areas of interest, like the lung and heart, are segmented with automated deep learning segmentation from the CXRs. The advancements in convolutional neural networks (CNN) have led to better performance in the image segmentation domain [8]. Multilayer CNNs [9] are used to detect different types of chest diseases and to segment medical tasks. A CNN-based automatic brain segmentation is performed on the MRI brain images to detect the tissues [10]. The authors of [11] proposed a semantic segmentation EG-CNN deep model to accurately detect the edges and boundaries of organs. Semantic segmentation is a pixel-wise classification that labels each pixel of a given class and groups the similar features. Medical images are complex, and this pixel-based semantic segmentation help with efficiently locating the infected areas in an image [12]. Due to low quality and pixel variations in the CXRs, the automatic semantic segmentation of the heart and other chest organs (lung and clavicle bones) is a challenging task. Previous studies addressed this issue and solved it by developing complex neural networks with higher computational resources [13]. 

In this study, we addressed this challenging issue and proposed a learning-based solution that performs accurate segmentation of lungs and heart to measure CTR from chest PA CXRs. CardioNet is the method proposed and used to determine the presence of cardiomegaly, and the graphical representation is discussed in Section 3. CardioNet is a semantic segmentation network that uses the dense identity features in the architecture to detect the edge properly within a few pixels. This deep model provides fine segmentation within fewer trainable parameters than the CNNs. CardioNet was trained on chest PA CXRs and provided the binary masks to compute the pixels and the positions of the anatomical organs. The semantic segmentation output and the calculated CTR decide the performance evaluation and make the final decision. 

The paper’s organization is as follows: We review the related work about cardiomegaly and the concept of chest anatomy segmentation based on the conventional handcrafted and deep features in Section 2. In Section 3, we propose a deep methodology for performing the semantic segmentation. Section 4 provides the CXRs data for the experiment and the data generation approach; the training and the testing of the proposed CardioNet model are performed in this section, followed by the experimental evaluation results. The work concludes with challenges in Section 5.

## 2. Related Works

There are two methods of segmenting the chest-related organs: handmade and deep features. The handmade feature-based methods utilize various image-processing schemes, where in-depth features are the learned features that depend on deep learning-based semantic segmentation.

### 2.1. Chest Anatomy Segmentation Using Conventional Handmade Features 

Handmade features are based on general image-processing approaches to segmenting the chest anatomy from the background. Most of the local feature-based methods do not consider multiclass segmentation of CXRs; rather, they focus on lung region segmentation. Peng et al. presented the Hull-CPLM method to detect the lung region of interest (ROI). The segmentation requires prior preprocessing for coarse segmentation, where the principle curve method is used to refine segmentation [14]. Candemir et al. proposed a non-grid registration-based lung segmentation method that performs the task in three steps: content-based image retrieval, sift-flow modeling for deformable registration, and graph cut optimization for boundary refinement [15]. Jaeger et al. computed three masks of lung segmentation, a probabilistic lung shape model and a Log Gabor mask, where segmentation was obtained by averaging and thresholding for the diagnostic purpose [16]. 

Jangam et al. presented a hybrid segmentation scheme that utilized an optimized clustering approach to exclude the lung field from the background in CXR images [17]. Vital et al. introduced an automatic system for lung field segmentation. A wavelet enhances the CXR images, and in the second step, OTSU thresholding is combined with mathematical morphology. For the third step, the active contour method improves the performance [18]. Ahmad et al. proposed a Gaussian derivative filter that considered seven different orientations of top segment fields. The segmentation task combines the Gaussian derivative filter with fuzzy c-mean clustering and thresholding [19]. Pattrapisetwong et al. used an unsupervised method for lung region exclusion from the background. They used preprocessing to enhance the image contrast, and then a shadow filter was applied to enhance the outline of the lungs; finally, multilevel thresholding segmented the resultant lung region [20]. 

Li et al. used graph-based lung segmentation. In detail, the CXR images are divided into several subregions and each region’s saliency value, where the cubic spline interpolation is used to obtain fine, smoother boundaries of the lung region [21]. Chen et al. proposed a system that estimates the effusion volume. The segmentation was performed using a 2-D image processing scheme, similar to the Harris corner detector, for enhancement using a convolutional process with a 2 × 2 mask to detect the lung contour [22]. Dawoud presented an iterative framework for lung field segmentation. The method calculates the intensity and shape information, where the main segmentation is handled with iterative thresholding [23]. Saad et al. showed that the edge detection from Sobel, Prewitt, and Laplacian could segment the lung fields; however, the accuracy decreases owing to the image noise. Therefore, combining these edge detectors with morphological operators can produce better results for lung segmentation in CXRs [24]. Chondro et al. proposed a low-order adaptive lung segmentation method based on the growing region. The ROI was obtained by brick–block binarization and morphology, where the boundary refinement was measured by statistical region growing and graph cutting [25]. Chung et al. considered lung segmentation a prerequisite task for diagnosis. The segmentation of the lungs performed by the Bayesian active contour model is an iterative process for segmentation [26].

### 2.2. Chest Anatomy Segmentation Using Deep Feature (CNN)

Conventional handcrafted features are based on specific local intensities; thus, these methods cannot perform multiclass segmentation at once. The performance parameters change from image to image. Therefore, automatic deep feature methods are an alternative to handmade features for evaluation. Long et al. used the first learning method for the multiclass chest anatomy segmentation. The encode-decoder is used for critical learning of higher-order structures. The encoder part extracts the features, and the decoder performs upsampling operations to obtain the final segmented output [27]. An adversarial network is proposed by Dong et al. to estimate the CTR. The produced model creates the predicted domain-independent output mask [28]. Tang et al. developed a transfer learning approach for lung segmentation, and it consists of two main modules. The first module is crisscross attention-wise responsible for the enriched global contextual information, whereas the second module, image-to-image translation, is used for data augmentation [29]. 

Souza et al. used a learning method to segment the lung regions. The original images were divided into patches, and those patches were classified into lung and non-lung classes by a neural network [30]. Kalinovsky et al. modified the encoder–decoder-based SegNet model for lung segmentation and achieved 96.2% accuracy [31]. This method worked on the limitation of the original SegNet model. It used the max-pooling features to upsample the features maps in the decoding layers, the LF-SegNet method developed by Mittal et al. [13] to perform lung segmentation. The lung segmentation was performed and the proposed model was evaluated on two famous chest X-ray datasets, JSRT and MC, and achieved 98.73% and 95.10% accuracy, respectively. Liu et al. [32] proposed a U-Net segmentation model on the JSRT dataset to extract the lung regions, and DenseNet is used to segment the lungs.

Venkataramani et al. developed ContextNets for semantic segmentation to adapt the target domain with fewer images [33]. Frid-Adar et al. considered an important application of semantic segmentation to detect the clavicle bone positioning using Chest X-rays. The modified architecture used to segment the clavicle bones and the weights of VGG16 used in the encoder [34]. Oliveira et al. presented a transfer learning-based approach f chest-related organs segmentation. The approach consists of pre-trained networks for semantic segmentation as; U-Net, fully connected network, and SegNet [35]. Wang et al. considered the instance segmentation to segment multiclass chest organs using chest X-ray images with Mask-RCNN [36]. Dong et al. presented a generative adversarial network (GAN) for a semantic segmentation purpose using CXRs [37]. Jiang et al. developed a CNN-based VGG16 segmentation model with prior weight initialization with fewer data. [38]. Most of the semantic segmentation from the radiographic images is performed by the UNet and encoder-decoder architectures or by the different variants of the followings; however, currently, the recurrent neural network (RNN) is also used for the segmentation purpose in radiology. Stollenga et al. [39] presented a segmentation 3D LSTM-RNN deep model to extract the brain features automatically. Chen et al. [40] used a semantic segmentation approach to 3D images by combining the LSTM-RNN and U-Net architecture.

## 3. Methodology

### 3.1. Proposed CardioNet

**Figure 2:**
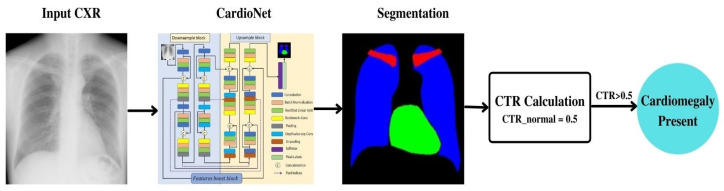
Flowchart of the proposed CardioNet methodology.

The flowchart of the proposed CardioNet model for the automatic semantic segmentation of chest-related organs is presented in Figure 2. An input chest of CXR images without any preprocessing is given to the CardioNet. CardioNet uses pixel-wise classification to segment the heart, lungs, and clavicle bones. The output of the model is the segmented masks for each class. The heart and lungs masks are utilized to calculate CTR to detect cardiomegaly, make accurate diagnostic decisions, and measure the model’s performance. The CardioNet is based on the dense connections between the downsample block, upsample block, and features boost block. Dense concatenation paths are used for the subsequent layers in the downsample and upsample block of the CardioNet and between the downsample and upsample block. The specific paths are introduced for the flow of information within the network to provide the edge information to the subsequent layers and the upsample block. Feature boost block is introduced to preserve minor features in the segmented image. It applies continuous convolutions without resizing the feature map size, which helps to premaintain needful spatial information to boost the segmentation performance. 

### 3.2. Chest Anatomy Segmentation Using CardioNet Architecture

Semantic segmentation in CXRs is not an easy task. X-ray images have inferior quality, whereas the correct CTR computation is based on the true lungs and heart boundaries. Moreover, the conventional semantic segmentation architectures [27,41] lack spatial information loss compensation as they are not using any residual or dense connectivity to empower the feature after the continuous convolutional operation. The proposed CardioNet is a robust network that incorporates three principal parts: a downsample block (DSB), an upsample block (USB), and a features boost block (FBB). 

**Figure 3:**
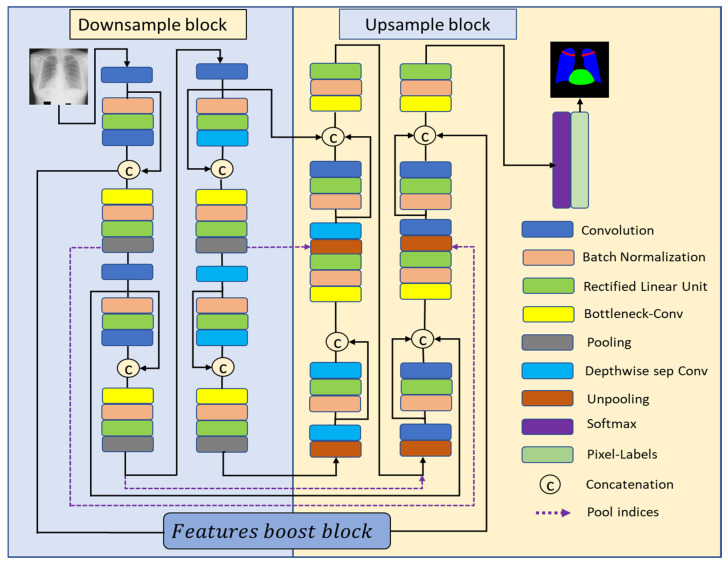
The proposed CardioNet architecture for CXR semantic segmentation.

Figure 3 shows the proposed CardioNet architecture for the CXR semantic segmentation. The DSB is an encoder part of the network that squeezes the vital information from the input images in combination with dense connectivity benefits. It consists of five 3 × 3 general convolutions and three depth-wise separable convolutions. The convolutional layers with many channels consume more trainable parameters. Therefore, the convolution in the deeper side of the DSB is replaced by depth-wise convolutions. The edge features in the CXR image can be very small, and those small features can be eliminated if the feature map size is greatly reduced within the network. It can be noticed from Table 1 that the smallest feature map size in the DSB is 21 × 21, which is not enough to represent the minor information available in the CXR; the minor features can be eliminated, and therefore, the FBB is used to apply continuous convolutions without resizing the feature map. The FBB retains the feature map on a flat feature map size of 350 × 350, which can contain the smaller and most valuable features. The USB is mainly based on three depth-wise separable convolutions combined with five 3 × 3 general convolutions and batch normalization. As mentioned previously, in the case of DSB, general convolutions with more channels are costly, so depth-wise separable convolutions are replacing those of the general convolution layers to reduce cost. Furthermore, softmax and pixel classification layers are used in the USB; where softmax layer works as the activation function and pixel classification layer provides a categorical label for each pixel in the image.

Moreover, the residual connections from the ResNet model were used to empower the features that degraded the contextual image information during the downsampling [41]. This problem is termed a vanishing gradient problem, and researchers attempted to address it using residual skip connections, but it still faces the information flow latency problem. We used the dense connectivity function from the DenseNet deep architecture to overcome this problem and concatenated the in-depth features [42]. DenseNet is a famous classification model that outperforms the previous networks while using fewer trainable parameters. The proposed architecture is entirely different from conventional networks such as the segmentation network (SegNet) [43], outer residual skip network (OR-Skip-Net) [44], and U-shaped network (U-Net) [45], which are deep neural networks with larger number of trainable parameters. The shallow architecture of CardioNet exhibits 1.72 million (M) trainable parameters, while the conventional networks such as SegNet, OR-Skip-Net, and U-Net, have 29.46 M, 09.70 M, and 31.03 M trainable parameters, respectively. The key architectural differences of the CardioNet with the conventional segmentation networks such as Seg-Net, OR-Skip-Net, and U-Net are listed in Table 2. 

**Figure 4:**
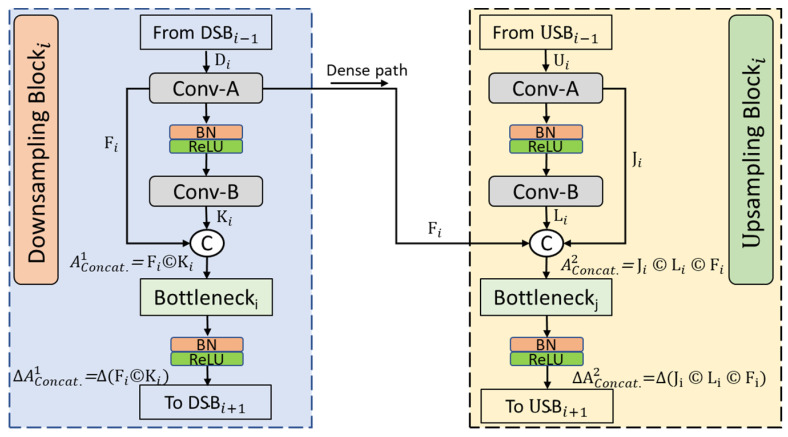
The CardioNet dense connectivity feature concatenation method.

Figure 4 illustrates the in-depth view of the proposed CardioNet with dense connectivity feature concatenation. CardioNet accomplishes the chest X-ray image; the spatial loss is compensated by concatenating deep, dense features. The dense concatenation method transfers these enriched features from the DSB to the USB, as shown in the figure. 

As shown in Figure 4, the first convolution layer in the downsample block, i.e., Conv-A, receives ${\mathrm{the}\;D}_{i}$ feature as input, and after applying the convolution operation, it outputs ${\mathrm{the}\;F}_{i}$ feature. This $F_{i}$ feature is rich with low-level spatial information, and it flows in two directions for concatenation. First, $F_{i}$ is provided to the second convolution (Conv-B), which converts into $K_{i}$. $K_{i}$ is the output achieved after the two convolution layers, and spatial loss of these convolutional operations is recovered by dense feature concatenation. The first aggregated rich, dense concatenated feature, $A_{\mathrm{Concat}.}^{1}$, is the output of $F_{i}$ and $K_{i}$ given by Equation (2):

(2) $$A_{\mathrm{Concat}}^{1}{=F}_{i}{\;©\;K}_{i}$$

Here © shows the depth-wise concatenation of ${\;F}_{i}$ and $K_{i}$. $F_{i}$ is also directly transferred to the corresponding USB to transfer the low-level spatial information to the last layers of the network for better performance. $A_{\mathrm{Concat}}^{1}$ is the resulting dense feature after the depth-wise concatenation. This process increases the number of filters for the dense features, the parameters, and the training time. To overcome this problem, we introduced the Bottleneck_i_ layer to decrease the number of filters with the combination of the BN and ReLU layers. The obtained feature after the Bottleneck_i_ block is ${\Delta A}_{\mathrm{Concat}}^{1}$ and given by Equation (3):

(3) $${\Delta A}_{\mathrm{Concat}.}^{1}{=\Delta (F}_{i}{\;©\;K}_{i})$$

Here, Δ represents the BN and ReLU operations in combination with the Bottleneck_i_ layer that limit the filters. Similarly, Figure 4 shows that each USB receives ${\mathrm{the}\;U}_{i}$ feature as input, and after applying the first convolution (Conv-A), it outputs ${\mathrm{the}\;J}_{i}$ feature. $J_{i}$ has less spatial loss than the next convolution layer, and it flows in two directions for concatenation. First, $J_{i}$ is provided to the second convolution (Conv-B), producing $L_{i}$. $L_{i}$ is the output obtained after two convolution operations that concatenate with $J_{i}$ from the first convolution (Conv-A) and ${\;F}_{i}$ from the corresponding DSB, creating a second aggregated rich feature $A_{\mathrm{Concat}.}^{2}$ given by Equation (4):

(4) $$A_{\mathrm{Concat}.}^{2}{=J}_{i}{\;©\;L}_{i}{\;©\;F}_{i}$$

Here, © shows the depth-wise concatenation of ${\;J}_{i}$, $L_{i}$, and $F_{i}$. $A_{\mathrm{Concat}.}^{2}$ is a powerful feature that contains rich information from the initial layers, resulting in better segmentation of the lung and heart region pixels. Again, to overcome the filter size problem, the Bottleneck_j_ block was used with a combination of BN and ReLU operations. ${\Delta A}_{\mathrm{Concat}.}^{2}$ is a bottleneck feature and is given by Equation (5):

(5) $${\Delta A}_{\mathrm{Concat}.}^{2}{=\Delta (J}_{i}{\;©\;L}_{i}{\;©\;F}_{i})$$

Here, Δ represents the combination of BN and ReLU operations with the Bottleneck_j_ layer limiting the filter size. Both the bottleneck features (${\Delta A}_{\mathrm{Concat}.}^{1}$, ${\Delta A}_{\mathrm{Concat}.}^{2}$) achieved from the downsample block or upsample block empowered the dense connectivity. The output $A_{\mathrm{Concat}.}^{2}$ of the last USB provided the Softmax and pixel classification.

**Figure 5:**
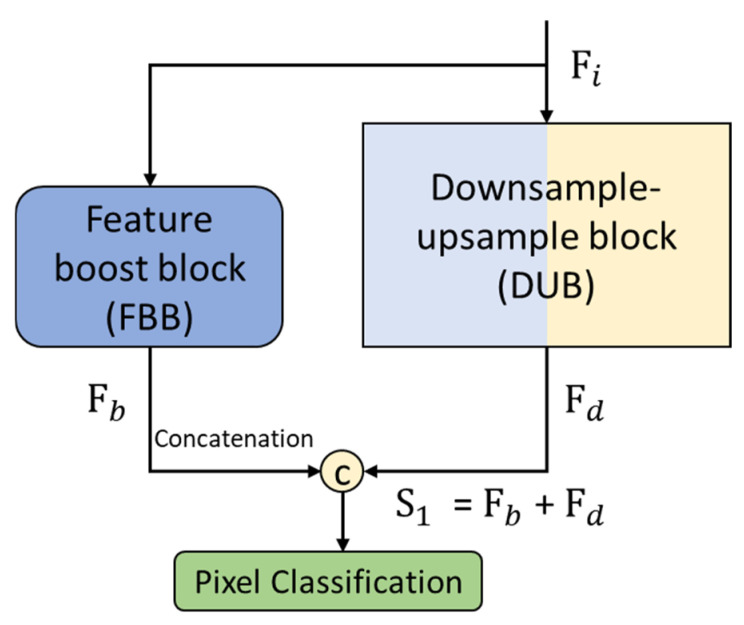
CardioNet features boost block concatenation.

The layer details in the booster block are shown in Table 3. The connectivity of the booster block in CardioNet and the dense feature aggregation principle are shown in Figure 5. The downsample–upsample block (DUB) takes the input feature, and this feature passes through several convolutional layers to extract the meaningful features for chest anatomy segmentation. This DUB provides the $F_{d}$ feature. $F_{d}$ is densely aggregated with the rich $F_{b}$ from the feature booster block (FBB). The features in FBB are without an extensive pooling operation and therefore contain the features that represent the low-level boundary information. Both $F_{d}$ and $F_{b}$ are aggregated with depth-wise concatenation to create $S_{1},$ given by Equation (6):

(6) $$S_{1}=F_{b}{\;©\;F}_{d}$$

Here, $S_{1}$ is the densely aggregated feature created by the depth-wise concatenation of $F_{d}\;$(feature coming from the DUB block) and $F_{b}$ (coming from FBB), representing the depth-wise concatenation.

## 4. Experiments

### 4.1. Data Augmentation

**Figure 6:**
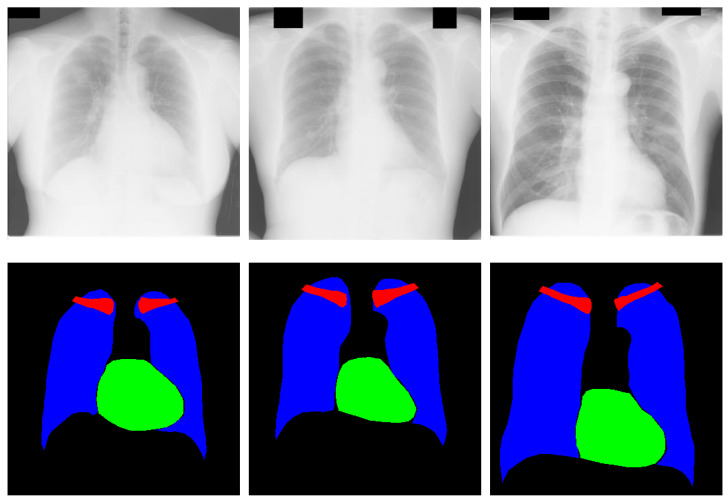
Sample CXR images from the JSRT dataset with corresponding ground truth.

We investigated chest anatomy segmentation in multiple classes. To analyze the performance of CardioNet, the Japanese Society of Radiological Technology (JSRT), i.e., a publicly available multiclass dataset [46], is used. In the JSRT dataset, there are 247 chest radiograph images for the lungs, heart, and clavicle, each annotated at the pixel level. A total of 154 of the 247 images show nodules, whereas the remaining 94 do not. The pixel space of each image in the dataset is 0.175 mm, and the image size is 2048 × 2048 pixels. The provided data are divided into two folds with odd (123) and even (124) numbered images. Training is done one-fold, testing one on the other and vice versa. Considering the two-fold cross-evaluation criteria, the results are obtained by averaging both folds. Figure 6 shows sample chest radiograph images from the JSRT dataset with corresponding ground truth. In detail, the red, green, and blue pixels show the clavicle bone, heart, and lungs, respectively.

**Figure 7:**
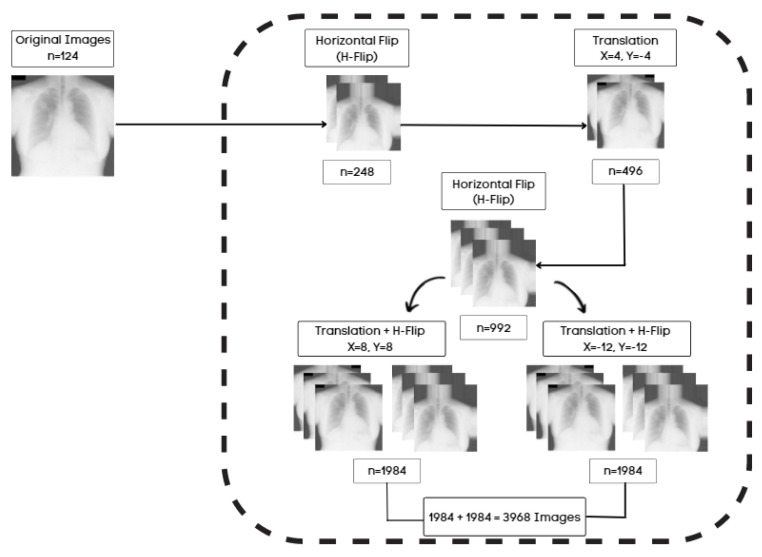
The proposed data augmentation approach increases the data size; the horizontal flip is represented as H-Flip.

CardioNet is a pixel-wise classification network that requires large training data to classify the multiple classes. The original image size and labels of 2048 × 2048 pixels of JSRT are resized to 350 × 350 pixels to reduce the memory usage of the graphic processing unit (GPU) and the training time. Different data generation approaches are applied to increase the data size so the model will learn accurately. Acquiring enough medically labeled data is difficult, requiring an expert to label the data. The training of CardioNet is performed with various images, and images are generated with data augmentation. The image transformations create artificial images by cropping, horizontal flipping, translation, and horizontal flipping with translation. Figure 7 represents the proposed data augmentation for this task. The first step of augmentation, horizontal flipping (H-Flip), is applied to 124 original images, which results in 248 images. The 248 images are translated (X = 4, Y = −4) to produce 496 images. In the third step, horizontally flipping the 496 images makes 992 images. Using the 992 images from the previous step, the next step applies the translation of (X = 8, Y = 8) with an H-Flip and the translation of (X = −12, Y = −12) with an H-Flip, resulting in 1984 and 1984 images, respectively. Consequently, 3968 images (1984 + 1984) are obtained and used as training images by combining transformational images from step four.

In our experiments, CardioNet is trained from scratch using MATLAB R2021b [47] (without fine-tuning or a pre-trained model, such as DenseNet, ResNet, Inception, or GoogleNet). CardioNet is trained and tested on a desktop computer, i.e., Intel^®®^ Core™ i7-8700 CPU. The system clock speed is 3.20 GHz, memory (RAM) is 16 GB, and NVIDIA GPU GeForce RTX 2060 (graphics memory of 8 GB) [48]. 

### 4.2. CardioNet Training

CardioNet contains several dense paths that allow the network to provide encoder–decoder connections both internally and externally, which is a useful tool to help the network converge quickly. As a result of the spatial edge information from the dense connectivity concatenation feature, the edges can be segmented finely without preprocessing. CardioNet training takes place from scratch, without any weight transfer or initialization. Since we designed CardioNet, fine-tuning is not required when training it.

**Figure 8:**
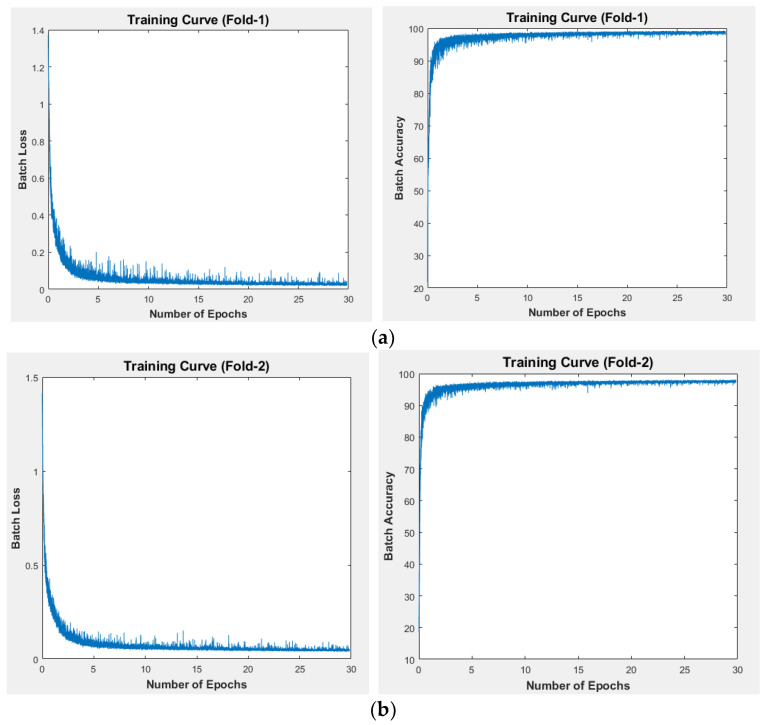
The training loss curves are on the left, and the training accuracy curves are on the right sides of the figures. The two-fold training is d on the number of epochs: (**a**) first fold, (**b**) second fold.

The training of CardioNet involves the following considerations. The gradient of the network is optimized with a well-known training optimizer called Adam. Adam can efficiently scale gradients diagonally, is suitable for large datasets, and can even handle moving object classification problems [49]. Adam is adopted as an optimizer for CardioNet training due to its benefits. Considering all other parameters, the learning rate of 0.001 for 30 epochs (51,660 iterations) is used throughout the CardioNet training. CardioNet requires high memory, so a small batch size with 4 images is considered. To maintain the gradient threshold, the global L2 normalization method with an epsilon of 0.000001 is used during the training. The cross-entropy loss function with median frequency is used to make training faster and similar to frequency balancing and cross-entropy [43,44,50,51]. Figure 8a,b represent the relationships between training loss and training accuracy for the 2-across-validation. The loss curves are on the left side and the accuracy curves of training are on the right side of the figures. The epochs are represented on the *x*-axis.

### 4.3. CardioNet Testing

#### 4.3.1. Chest Anatomy Segmentation Testing Using CardioNet

A CXR image without data preprocessing is given to CardioNet as an input image. This input image is passed through the CardioNet downsample block and upsample block to acquire the segmented output. The final output of the proposed network is a multiclass segmentation mask. These segmented masks further evaluate the final output by generating the chest-related automatic semantic segmentation results. The performance of CardioNet is evaluated and measured using different metrics. The following segmentation metrics are calculated: Accuracy (Acc), Jaccard index (J), and Dice coefficient(D). J is a mean intersection over union (IoU), and D is calculated as [1,36,52] for a JSRT dataset. Equations (7)–(9) give the formulas for evaluation metrics:

(7) $$\mathrm{Acc}=\frac{\mathrm{TP}+\mathrm{TN}}{\mathrm{TP}\;+\;\mathrm{FP}\;+\mathrm{FN}+\mathrm{TN}}$$

(8) $$J=\frac{\mathrm{TP}}{\mathrm{TP}\;+\;\mathrm{FP}\;+\mathrm{FN}}$$

(9) $$D=\frac{2\mathrm{TP}}{P\;+\;\mathrm{FP}\;+\mathrm{FN}}$$

Here, TP = true positives, FP = false positives, and FN = false negatives. We are considering multiclass segmentation, considering lung class as an example: TP and FP are predicted as lung pixels and non-lung pixels in the ground truth, respectively. TP pixels are the pixels that are predicted as lung pixels and listed as lung pixels in the ground truth. FN are those lung pixels with ground truth predicted as non-lung pixels by our CardioNet model. 

**Figure 9:**
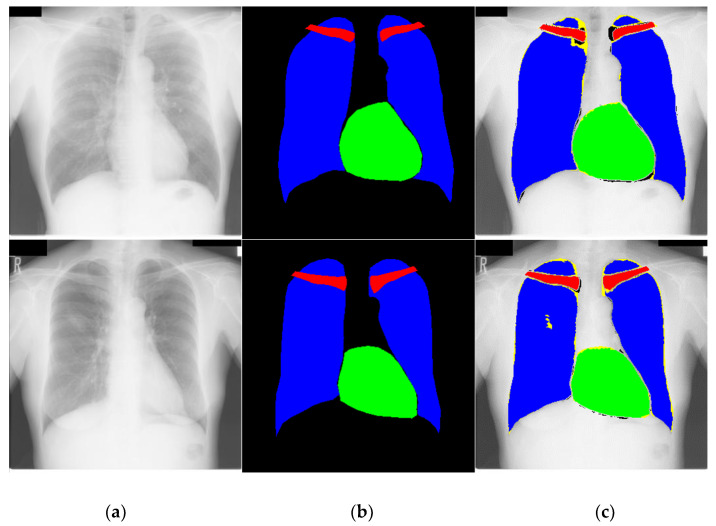
Examples of chest-related organs by CardioNet: (**a**) original chest PA X-ray image; (**b**) image with a ground-truth mask; (**c**) CardioNet predicted mask.

The segmented chest X-ray images with CardioNet using the JSRT dataset are shown in Figure 9. The FP convention is black; the FN convention is yellow; and the TP convention is shown in green, blue, and red for the heart, lung, and clavicle bone classes, respectively. While there are some bad segmentation cases, there is no significant segmentation error using our method for the test images.

#### 4.3.2. Ablation Study

An ablation study of our proposed method was conducted to prove the efficiency of CardioNet. We considered two variants of CardioNet, one with the booster block, i.e., CardioNet-B (mentioned as CardioNet throughout the whole manuscript), and the second one without the booster block, referred to as CardioNet-X. Here, we compared CardioNet-B with CardioNet-X. The experiments showed that the effectiveness of the booster block in CardioNet-B provides superior results to those of CardioNet-X (without booster block). Table 4 shows that CardioNet-B with the booster block provides higher Acc, J, and D values than CardioNet-X with a minor increase in the number of trainable parameters. Booster block creates considerable performance differences owing to preserving spatial information to boost the segmentation performance.

#### 4.3.3. Comparison of CardioNet with Deep Methods

This section compares CardioNet segmentation performance with other methods. The segmentation performance of the existing state-of-the-art methods compared with CardioNet is shown in Table 5. This comparison is based on the JSRT dataset. The local feature-based methods and the learned feature-based methods are presented separately in Table 5. Based on J and D, the study results demonstrate that CardioNet outperforms other studies for chest anatomy segmentation.

#### 4.3.4. Lung Segmentation from MC Dataset Using CardioNet

**Figure 10:**
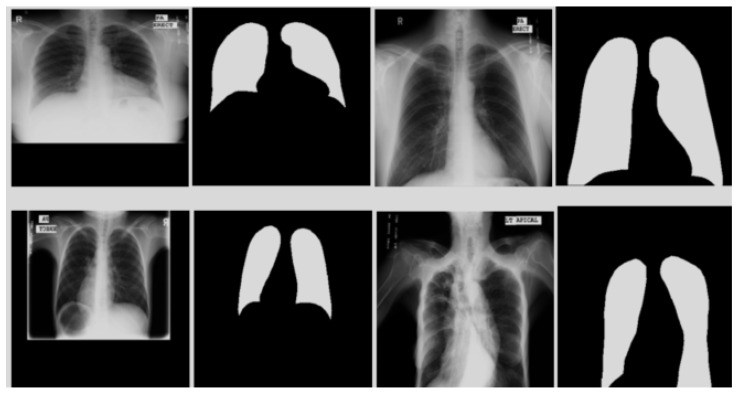
Examples of MC chest X-ray images with ground truths.

**Figure 11:**
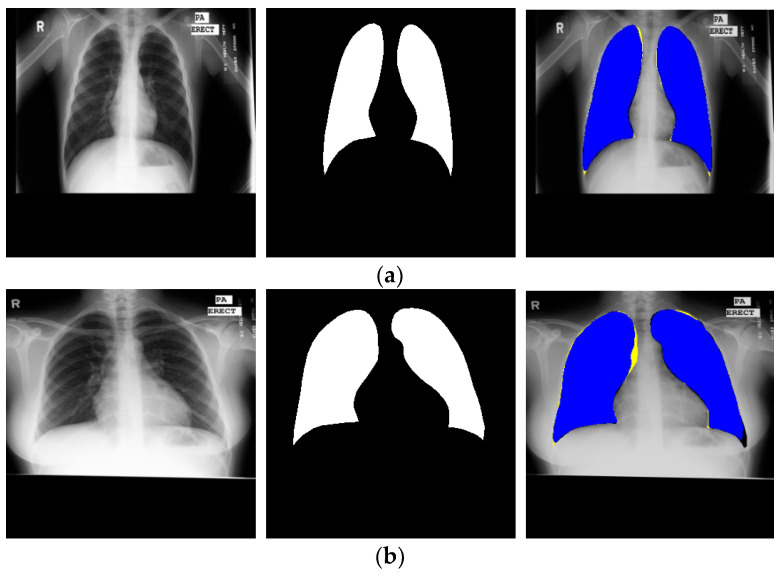
Examples of CardioNet lung semantic segmentation for MC data: (**a**) represents the best and most accurate segmentation (right image) and (**b**) shows the worst lung segmentation (right image) by proposed CardioNet.

After obtaining the valuable results from the JSRT image dataset, we evaluated the performance of our CardioNet model with the Montgomery County chest X-rays (MC) dataset. MC is a publicly available dataset published by the famous Montgomery County tuberculosis program, one of the most famous in the USA. It has 138 chest PA X-ray images with 80 normal and 58 TB cases. Figure 10 shows sample images from the MC data with lung contour binary masks, and the format of the X-ray images format is PNG. We divided the MC dataset into training and testing following [60], where the training set has 80 images, the validation set has 20, and the testing set consists of 38 images. The MC dataset is also augmented, and the image size was artificially increased using the same augmentation approach used for the JSRT dataset (Section 4.1). We only provided the ground truth masks for lungs. The CardioNet results for the MC dataset are shown in Figure 11. However, the experimental comparison of CardioNet with other existing models is shown in Table 6. The experimental results clearly show that CardioNet outperformed for lung segmentation and can be used in real-world medical applications for diagnostic purposes.

#### 4.3.5. Automated Computation of CTR by the Proposed Method

**Figure 12:**
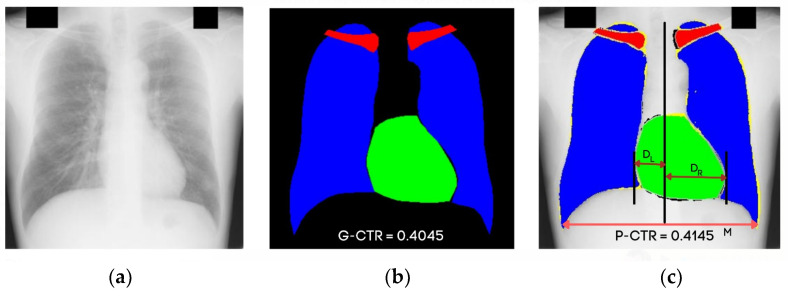
Example image from the JSRT dataset for calculating CTR using the proposed CardioNet: (**a**) original image; (**b**) ground truth image annotated with the cardiothoracic ratio (G-CTR); (**c**) predicted mask annotated with the cardiothoracic ratio (P-CTR).

As explained in the introduction section, medical practitioners can use CTR in diagnosing cardiomegaly, a heart enlargement disease. CTR is a quantitative measure of heart enlargements in CXRs to detect cardiomegaly and boundaries of other chest organs. CTR estimates the size of the heart. The cardiac health experts manually calculate it. Our proposed semantic segmentation network (CardioNet) can automate the computation of CTR by accurately segmenting the lungs and heart boundaries. As shown in Figure 12a, the heart boundary is critical in X-ray images due to a change in pixel values. The CTR can only be calculated by the proposed method once the heart and lungs are segmented properly. With our feature-boosting mechanism, we can achieve accurate boundaries of the heart and lungs even with minor changes in pixel values. 

An example image from the JSRT dataset for calculating the CTR of the ground truth and the predicted mask (by CardioNet) using Equation (1) is shown in Figure 12b,c, respectively. For calculating the CTR, we need the ratio of distance $D_{L}+{\;D}_{R}$ and M. Here, $D_{L}+{\;D}_{R}$ is the distance between two extreme points of the heart. M is the maximum horizontal distance between two extreme outer points for both lungs. The distance $D_{L}+{\;D}_{R}$ by the predicted mask is 126 pixels, and the distance M is 304. Hence, using Equation (1), the predicted CTR (P-CTR) will be 0.4145. On the other hand, the distance D_L_ + D_R_ by the ground truth mask is 123 pixels, and the distance M is 303. Therefore, the ground truth CTR (G-CTR) calculated by Equation (1) is 0.4045. In JSRT, masks are prepared under the supervision of an expert radiologist. It can be determined from the obtained results that the proposed automatic method has predicted the CTR very efficiently. Hence, it can aid the medical practitioner in diagnosing using CTR and other chest anatomy segmentation as an alternative system.

## 5. Conclusions

Anatomical chest structures (lungs, hearts, and clavicle bones) were segmented using a residual connection-based semantic segmentation network (CardioNet) for diagnostic purposes. Even under nonideal situations and multiple classes, the method provides excellent segmentation accuracy. Since the pixel value of the heart is low and its boundary edges blend with the lungs, segmenting the heart is essential. CardioNet can segment the heart accurately, even in X-ray images of inferior quality. The segmentation accuracy of the heart and lung regions is directly related to the CTR. Conventional CNNs reduce the feature map size to classify classes, resulting in the depreciation of the minor information (clavicle bone and small-sized heart) due to the overuse of the max-pooling layers. The feature map size is not reduced in the network for classification; fewer pooling layers are used, and a large feature size helps restore the minor classes’ information. The direct, outer, dense-connectivity feature concatenation causes direct information transfer, enabling CardioNet to converge faster, as seen in the training accuracy and loss curves. In our work, the proposed CardioNet automatically detects the boundaries of the lungs and heart to accurately calculate the CTR. Multiple cardiac and lung diseases can be diagnosed using the CTR. 

## Figures and Tables

**Table 1:** Cardio-Net with feature concatenation, where the Downsample and Upsample blocks include Convolution (Conv), Bottleneck convolution (Bottleneck-C), Depth-wise separable convolution (DW-Sep-Conv), Concatenation, and Pool. Batch normalization and ReLU layers are used with convolutions and denoted as “**”.

Block	Layer Name	Layer Size (Height × Width × Number of Channels), (Stride)	Filters/Groups	Output
Downsample block	Conv-1-1 **	3 × 3 × 64 (S = 1)	64	350 × 350 × 64
	Conv-1-2	3 × 3 × 64 (S = 1)	64	350 × 350 × 64
	Concatenation-1			350 × 350 × 128
	Bottleneck-C-1 **	1 × 1 (S = 1)	64	350 × 350 × 64
	Pool-1 2 × 2 (S = 2)			175 × 175 × 64
	Conv-2-1 **	3 × 3 × 64 (S = 1)	128	175 × 175 × 128
	Conv-2-2	3 × 3 × 128 (S = 1)	128	175 × 175 × 128
	Concatenation-2			175 × 175 × 256
	Bottleneck-C-2 **	1 × 1 (S = 1)	128	175 × 175 × 128
	Pool-2 2 × 2 (S = 2)			87 × 87 × 128
	Conv-3-1 **	3 × 3 × 128 (S = 1)	256	87 × 87 × 256
	DW-Sep-Conv-3-2	3 × 3 × 256 (S = 1)	256	87 × 87 × 256
	Concatenation-3			87 × 87 × 512
	Bottleneck-C-3 **	1 × 1 (S = 1)	128	87 × 87 × 256
	Pool-3 2 × 2 (S = 2)			43 × 43 × 256
	DW-Sep-Conv-4-1 **	3 × 3 × 256 (S = 1)	256	43 × 43 × 256
	DW-Sep-Conv-4-2	3 × 3 × 256 (S = 1)	256	43 × 43 × 256
	Concatenation-4			43 × 43 × 512
	Bottleneck-C-4 **	1 × 1 (S = 1)	256	43 × 43 × 256
	Pool-4 2 × 2 (S = 2)			21 × 21 × 256
Upsample block	UnPool-4 2 × 2 (S = 2)			43 × 43 × 256
	DW-Sep-Conv-4-2 **	3 × 3 × 256 (S = 1)	256	43 × 43 × 256
	DW-Sep-Conv-4-1	3 × 3 × 256 (S = 1)	256	43 × 43 × 256
	Concatenation-5			43 × 43 × 512
	Bottleneck-C-5 **	1 × 1 (S = 1)	256	43 × 43 × 256
	UnPool-3 2 × 2 (S = 2)			87 × 87 × 256
	DW-Sep-Conv-3-2 **	3 × 3 × 256 (S = 1)	256	87 × 87 × 256
	Conv-3-1	3 × 3 × 256 (S = 1)	128	87 × 87 × 128
	Concatenation-6			87 × 87 × 640
	Bottleneck-C-6 **	1 × 1 (S = 1)	128	87 × 87 × 128
	UnPool-2 2 × 2 (S = 2)			175 × 175 × 128
	Conv-2-2 **	3 × 3 × 128 (S = 1)	128	175 × 175 × 128
	Conv-2-1	3 × 3 × 128 (S = 1)	64	175 × 175 × 64
	Concatenation-7			175 × 175 × 320
	Bottleneck-C-7 **	1 × 1 (S = 1)	64	175 × 175 × 64
	UnPool-1 2 × 2 (S = 2)			350 × 350 × 64
	Conv-1-2 **	3 × 3 × 64 (S = 1)	64	350 × 350 × 64
	Conv-1-1	3 × 3 × 64 (S = 1)	64	350 × 350 × 64
	Concatenation-8			350 × 350 × 160
	Bottleneck-C-8 **	1 × 1 (S = 1)	2	350 × 350 × 2

**Table 2:** An architectural comparison of CardioNet with famous segmentation methods.

Method	Other Architectures	CardioNet
SegNet [43]	26 convolutional layers (3 × 3)	16 convolutional layers (3 × 3)
	No depth-wise separable convolution	6 depth-wise separable convolutions are involved in reducing the number of trainable parameters
	No skip connections are used.	Dense skip paths are used.
	Each block has a different number of convolutional layers	Each block has the same number of convolutions (2 convolutions)
	No booster block	Booster block.
	5 pooling layers	4 pooling layers
	The number of trainable parameters is 29.46 M.	The number of trainable parameters is 1.72 M.
OR-Skip-Net [44]	There is no internal connectivity between the convolutional layers in the encoder and decoder.	Both internal and external connectivities are used.
	Residual connectivity is used.	Dense connectivity is used.
	16 convolutional layers (3 × 3)	16 convolution layers (3 × 3) including 6 layers of booster block (max. depth 32)
	No depth-wise separable convolution	6 depth-wise separable convolution is involved in reducing the number of trainable parameters
	Bottleneck layers are not used.	Bottleneck layers are used to reduce the number of channels.
	The number of trainable parameters is 09.70 M	The number of trainable parameters is 1.72 M
U-Net [45]	23 convolutional layers are used	16 convolutional layers (3 × 3)
	No depth-wise separable convolution	6 depth-wise separable convolution is involved in reducing the number of trainable parameters
	Up convolutions are used in the expansive part for upsampling	Unpooling layers are used for upsampling
	External dense connectivity is used from encoder to decoder.	Both internal and external dense connectivity in downsampling and upsampling block
	Cropping is required owing to border pixel loss during convolution	Cropping is not required
	The number of trainable parameters is 31.03 M	The number of trainable parameters is 1.72 M

**Table 3:** Feature map size details for features boost block. Batch normalization and ReLU layers are used with bottleneck convolution layers as a unit and denoted as “**”. Stride is 1 throughout the features boost block.

Block	Layer Name	Layer Size (Height × Width × Number of channels)	Filters/Groups	Output
Features boost block (FBB)	Bottleneck-C **	1 × 1 × 8	8	350 × 350 × 8
	Boost-Conv-1-1 **	3 × 3 × 8	8	350 × 350 × 8
	Boost-Conv-1-2 **	3 × 3 × 8	8	350 × 350 × 8
	Boost-Conv-2-1 **	3 × 3 × 16	16	350 × 350 × 16
	Boost-Conv-2-2 **	3 × 3 × 16	16	350 × 350 × 16
	Boost-Conv-3-1 **	3 × 3 × 32	32	350 × 350 × 32
	Boost-Conv-3-2 **	3 × 3 × 32	32	350 × 350 × 32

**Table 4:** The ablation study-based comparison of CardioNet-B and CardioNet-X on the JSRT dataset.

Methods	Segmentation Regions	Number of Trainable Parameters	Number of 3 × 3 Convolution Layers	Acc	J	D
CardioNet-X	Lungs	1.57 M	10	98.08	93.04	96.38
	Heart			98.91	88.70	93.84
	Clavicle bone			97.81	85.99	91.53
CardioNet-B	Lungs	1.72 M	16	99.24	97.28	98.61
	Heart			99.08	90.42	94.76
	Clavicle bone			99.76	86.74	92.74

**Table 5:** Accuracies of CardioNet and existing methods for the JSRT dataset (unit: %).

Type	Method	Lungs			Heart			Clavicle Bone		
		Acc	J	D	Acc	J	D	Acc	J	D
Local feature-based methods	Coppini et al. [53]	-	92.7	95.5	-	-	-	-	-	-
	Jangam et al. [17]	-	95.6	97.6	-	-	-	-	-	-
	ASM default [54]	-	90.3	-	-	79.3	-	-	69.0	-
	Chondro et al. [25]	-	96.3	-	-	-	-	-	-	-
	Candemir et al. [15]	-	95.4	96.7	-	-	-	-	-	-
	Dawoud [23]	-	94.0	-	-	-	-	-	-	-
	Peng et al. [55]	97.0	93.6	96.7	-	-	-	-	-	-
	Wan Ahmed et al. [19]	95.77	-	-	-	-	-	-	-	-
Deep feature-based methods	Dai et al. FCN [56]	-	94.7	97.3	-	86.6	92.7	-	-	-
	Oliveira et al. FCN [35]		95.05	97.45		89.25	94.24		75.52	85.90
	OR-Skip-Net [44]	98.92	96.14	98.02	98.94	88.8	94.01	99.70	83.79	91.07
	ResNet101 [36]		95.3	97.6		90.4	94.9		85.2	92.0
	ContextNet-2 [33]	-	96.5	-			-	-	-	-
	BFPN [52]	-	87.0	93.0	-	82.0	90.0	-	-	-
	InvertedNet [1]		94.9	97.4		88.8	94.1		83.3	91.0
	HybridGNet [57]			97.43			93.34			
	RU-Net [58]					85.57				
	MPDC DDLA U-Net [59]		95.61	97.90						
	**CardioNet** **(Average of Fold 1** **and Fold 2)**	**99.24**	**97.28**	**98.61**	**99.08**	**90.42**	**94.76**	**99.76**	**86.74**	**92.74**

**Table 6:** The comparison of proposed CardioNet and other state-of-the-art methods for the MC dataset (unit: %).

Type	Method	Accuracy	Jaccard Index	Dice Coefficient
Handcrafted feature-based methods	Vajda et al. [61]	69.0	-	-
	Candemir et al. [4]	-	94.1	96.0
	Peng et al. [55]	97.0	-	-
Deep feature-based methods	Feature selection and Vote [62]	83.0	-	-
	Feature selection with BN [62]	77.0	-	-
	Bayesian feature pyramid network [52]	-	87.0	93.0
	Souza et al. [30]	96.97	88.07	96.97
	HybridGNet [57]			95.4
	MPDC DDLA U-Net [59]		94.83	96.53
	**CardioNet (proposed method)**	**98.92**	**95.61**	**97.75**

## Data Availability

Not applicable.
